# Assessment of ‘Accredited Social Health Activists’—A National Community Health Volunteer Scheme in Karnataka State, India

**Published:** 2015-03

**Authors:** Farah N. Fathima, Mohan Raju, Kiruba S. Varadharajan, Aditi Krishnamurthy, S.R. Ananthkumar, Prem K. Mony

**Affiliations:** ^1^Department of Community Health, St. John's Medical College, Bangalore, India; ^2^Karnataka State Health Systems Resource Centre, Bangalore, India; ^3^Division of Epidemiology and Population Health, St. John's Research Institute, Bangalore, India

**Keywords:** Assessment, Community health volunteer, India

## Abstract

About 700,000 Accredited Social Health Activists (ASHA) have been deployed as community health volunteers throughout India over the last few years. The objective of our study was to assess adherence to selection criteria in the recruitment of ASHA workers and to assess their performance against their job descriptions in Karnataka state, India. A cross-sectional survey, using a combination of quantitative and qualitative methods, was undertaken in 2012. Three districts, 12 *taluks* (subdistricts), and 300 villages were selected through a sequential sampling scheme. For the quantitative survey, 300 ASHAs and 1,800 mothers were interviewed using sets of structured questionnaire. For the qualitative study, programme officers were interviewed via in-depth interviews and focus group discussions. Mean±SD age of ASHAs was 30.3±5.0 years, and about 90% (261/294) were currently married, with eight years of schooling. ASHAs were predominantly (>80%) involved in certain tasks: home-visits, antenatal counselling, delivery escort services, breastfeeding advice, and immunization advice. Performance was moderate (40-60%) for: drug provision for tuberculosis, caring of children with diarrhoea or pneumonia, and organizing village meetings for health action. Performance was low (<25%) for advice on: contraceptive-use, obstetric danger sign assessment, and neonatal care. This was self-reported by ASHAs and corroborated by mothers. In conclusion, ASHA workers were largely recruited as per preset selection criteria with regard to age, education, family status, income, and residence. The ASHA workers were found to be functional in some areas with scope for improvement in others. The role of an ASHA worker was perceived to be more of a link-worker/facilitator rather than a community health worker or a social activist.

## INTRODUCTION

Most countries with high disease burden in southern Asia and sub-Saharan Africa are not on track to achieving the Millennium Development Goals (MDG) on maternal and child health and nutrition by the year 2015 (1). India, accounting for 17% of the world's population, contributes to 19% of global maternal deaths and 21% of global childhood deaths (2–4). It has, however, made significant strides in improving maternal and child healthcare coverage over the last decade, especially after launching the National Rural Health Mission (NRHM) programme since 2005 (5). This includes an explicit commitment to increasing public spending on health from 0.9% to 2-3% of Gross Domestic Product (GDP) by the Government of India. The NRHM comprises a combination of several strategies/schemes namely—a conditional cash transfer scheme [*Janani Suraksha Yojana* (JSY)] for institutional deliveries; an emergency transport mechanism (Call Ambulance ‘108’ initiative of Emergency Management and Research Institute; improved communitization through constitution of Village Health, Sanitation and Nutrition Committees (VHSNC), and investments in health infrastructure and health manpower, including the creation of a new cadre of community health volunteers [Accredited Social Health Activists (ASHA)] across all villages in India (5–7).

Community health volunteer schemes are considered vital to achieving the goal of increasing community participation and access to the healthcare system (8–10). An ASHA is a woman selected by the community, resident in the community and who is trained, deployed, and supported to function in her own village to improve the health status of the people through securing their access to healthcare services. Her job responsibilities are three-fold, including the role of a link-worker (facilitating access to healthcare facilities and accompanying women and children), that of a community health worker (depot-holder for selected essential medicines and responsible for treatment of minor ailments), and of a health activist (creating health awareness and mobilizing the community for change in health status) (5–9). Till date, 700,000 ASHA workers have been trained and deployed across the country. Being lay workers, the governance structure, including their selection, incentives, and community ownership, and their performance in health and community development, have been identified as critical issues that need to be monitored. It is, therefore, imperative to know what kind of ASHA workers are in place and what work they are doing. This has implications for the role/utility of community health workers in several settings where health systems are traditionally weak. Studies in other parts of the country have found issues with the ASHA recruitment process. Further, there has also been lack of clarity on the performance and role of the ASHA workers (5,10). The objective of this study was to assess adherence to selection criteria in the recruitment of ASHA workers and to evaluate the performance of ASHA workers in the state of Karnataka in southern India.

## MATERIALS AND METHODS

### Study setting

The study was carried out in Karnataka state, India. It has a population of 61 million, life expectancy of 65 years, female literacy rate of 58%, sex ratio of 973 females per 1,000 males, and 61% rural population (11), with an overall Human Development Index of 0.65 (12). The maternal mortality ratio was 178 per 100,000 livebirths (period 2007-2009), and infant mortality ratio was 38 per 1,000 livebirths (year 2011) while total fertility rate was 2.0 (13).

### Study design

We employed a cross-sectional epidemiological study design for this enquiry and used a mixed-methods approach, with a combination of quantitative and qualitative research methods (14).

### Sampling technique and sample-size

We adopted the multistage sampling design recommended by the National Health Systems Resources Centre (NHSRC) at the Ministry of Health and Family Welfare, New Delhi, for the evaluation of ASHA programme in India. A sequential sampling scheme was followed, with selection of districts and by *taluks* (subdistricts) and lastly by villages (9).

Selection of districts was done by purposive sampling technique (15) based on the perception of the state-level chief ASHA programme officer who chose three districts in one of the two following ways: (i) district(s) that had a large proportion of socioeconomically-disadvantaged populations (Kolar and Chamrajanagar districts were selected because of substantial proportions of scheduled caste and scheduled tribe (SC/ST) populations) and (ii) a district that had implemented a recent programmatic innovation (Haveri district was selected because of the recent introduction of electronic payment of honouraria to the bank accounts of ASHA workers in the district).

Four *taluks* were selected from within each district by quota sampling (15) based on the relative performance on health programmes as perceived by state/district health department officials (from two well-performing *taluks* and two relatively not-so-well-performing *taluks*). A total of 12 such *taluks* were included in the study.

A cluster was defined as a health subcentre, the peripheral health outpost in the public sector's multi-tier health system. Twenty-five subcentres were selected from among all subcentres in the four *taluks* of each district through systematic random sampling with population proportionate-to-size (16). Within each cluster, the headquarter village of the subcentre (defined as ‘index village’) was selected, along with three other nearby villages within a radius of 5-10 km. Thus, a total of 300 villages (100 in each district) were selected.

### Ethical clearance

Ethical approval was obtained from the Institutional Ethics Review Board of St. John's Medical College, Bangalore, India.

### Sample subjects

#### Quantitative survey

There were three categories of respondents for the quantitative survey (9). One ASHA was selected from each village included in the study sample, making up a total of 300 ASHAs from the state. In addition, two sets of beneficiaries were interviewed in each village: four recently-delivering mothers, with children aged 0-6 month(s), to study utilization of obstetric care services and two ‘older’ mothers, with children aged 6-24 months, with any illness in the preceding month, to study utilization of childcare services. A total of 1,200 ‘recent’ mothers and 600 ‘older’ mothers were, thus, interviewed.

For a random-sampling design, the required sample-size for estimating a proportion of 50% with an absolute precision of 10% will be 96. This is the highest sample required for all proportions between 0 and 100 (17). We assumed a 50% prevalence for self-reported performance of a health task by an ASHA, with an absolute precision of 10% and with 95% confidence and rounded off the required sample-size to 100 ASHAs in each district. Therefore, a total sample-size of 300 ASHAs in the state was estimated for the study. To account for clustering effect, the sample-size was re-calibrated by considering a design effect of 4 for the interview of recent mothers and a design effect of 2 for the interview of older mothers.

#### Qualitative survey

The participants for the qualitative component included ASHA programme officers at the state, district, and *taluk* (subdistrict) levels (9).

### Study instrument

For the quantitative survey, sets of structured questionnaire (9) developed by NHSRC were used for interviewing the ASHA workers and their beneficiaries. The questionnaire for ASHA workers captured information on their sociodemographic particulars, selection process, training received, and tasks performed while the beneficiary questionnaire captured details on the most recent pregnancy or illness in an under-five child and the role played by ASHA in the management of these conditions. These sets of questionnaire were translated into Kannada and back-translated into English. These were subsequently pretested for clarity of items and reliability prior to use in the survey.

A semi-structured fieldguide was developed for use in the qualitative study. We used key-informant in-depth interviews at the state level and a combination of in-depth interviews and focus group discussions at the district level. The themes covered were achievements and opportunities of the ASHA programme. They were also asked to grade the intended role/current performance of the ASHAs as a link-worker, as a community health worker, and as an activist.

### Study personnel

For the quantitative component, the study team consisted of district-level coordinators (3), field supervisors (4 in each district), and field investigators (16 in each district). The coordinators (FNF, KS, AK) were community health physicians. Supervisors and field investigators who had completed Bachelor's-level and secondary school-level education respectively underwent three days’ training in study methods and instruments.

For the qualitative component, state and district-level interviewers were researchers (FNF, KS, AK, PKM) with expertise in qualitative research methodology as well as maternal and child health programmes.

### Quality control, data management, and analysis

Data collection was undertaken during February-May 2012. Five percent of all sets of questionnaire collected in the quantitative survey were re-sampled for repeat survey by an independent field team of research assistants for validation and feedback. Data were entered and analyzed using SPSS (version 17) for the quantitative component; for the qualitative study, manual analysis according to major themes was undertaken. Adherence to selection criteria was studied with reference to Government of India's norms (9). Functionality or performance of ASHAs was measured against their job description by self-reported performance of key tasks by the ASHAs themselves and also as reported by the beneficiaries (9). Simple descriptive statistics are presented.

## RESULTS

### Quantitative study

The coverage of sampled villages was 100%; participant coverage was 98% (294/300) for ASHAs, 96% (1,156/1,200) and 97% (579/600) for ‘recent’ and ‘older’ mothers respectively.

### ASHA workers–adherence to selection criteria and functional performance

Table 1 depicts the sociodemographic characteristics of the ASHA workers. Nearly 95% (274/291) of them were in the age-group of 20-39 years; three-fourths (215/291) were in the age-group of 26-35 years, mean±SD age being 30.3±5.0 years. About 90% (261/293) were currently-married women. Most (73.1%) ASHAs had one or two child(ren), those with no children were 5.2%, and those with >2 children were 21.7%. Nearly 90% (264/294) of ASHAs had completed eight years of schooling. An overwhelming majority (97%) were Hindus, and nearly two-thirds belonged to socially-disadvantaged caste groups.

Majority of ASHAs (59%) were from households with income of 1,000 to 3,000 Indian Rupees (INR) (US$ 18-50) per month; about 16% were from households with income of <1,000 INR per month. For most ASHAs (78.4%), the chief earning member of the family was the husband. ASHA herself was the chief earning member in 11% of the households. About 44% of ASHAs reported that the main income-generating activity they performed was the ASHA's work itself. About half of the ASHAs participated in other income-generating activities, like agriculture, daily-wage labour, animal husbandry, etc.

**Table 1.** Sociodemographic characteristics of ASHA workers

Characteristics	Number*	Percentage	95% CI
1. Age (completed years) [N=291]			
▪20-29	136	46.7	40.3-51.7
▪30-39	138	47.4	41.6-53.1
▪≥40	17	5.8	3.1-8.5
▪Mean age (years)	30.3±5.0		29.7-30.9
▪Age-range (years)	20-47		
2. Marital status [N=293]		88.8	84.2-91.7
▪Married	261	10.9	7.3-14.4
▪Unmarried/Widowed/Divorced/Separated	32		
3. No. of children [N=290]		5.2	2.6-7.6
▪0	15	73.1	67.9-78.2
▪1-2	212	21.7	16.9-26.4
▪≥3	63		
4. Educational status [N=294]			
▪≤Grade 7	30	10.2	6.8-13.7
▪Grade 8 and above	264	89.8	86.3-93.2
5. Religion [N=294]			
▪Hindu	286	97.3	95.4-99.1
▪Other	8	2.7	0.8-4.5
6. Caste [N=288]			
▪SC/ST/OBC†	182	63.2	58.1-69.2
▪Other	106	36.8	31.2-42.3
7. Main income source [N=268]			
▪ASHA's work	119	44.4	38.4-50.3
▪Other	149	55.6	49.1-61.5
8. Chief earning member in family [N=283]			
▪Self	31	11	7.3-14.6
▪Husband/Other	252	89	85.3-92.6
9. Total household income (INR per month)‡ [N=292]			
▪<1,000	48	16.4	12.1-20.6
▪1,000-3,000	173	59.2	53.5-64.8
▪>3,000	71	24.3	19.3-29.2

*Total does not add up to 294 because of missing values

†SC/ST/OBC=Scheduled caste/Scheduled tribe/Other backward castes

‡INR=Indian Rupees (60 INR=1 US$)

Table 2 shows the proportion of ASHA workers engaged in various tasks over the preceding six months. A very high proportion (>80%) of them had reported key activities, such as home-visits, ANC counselling, and escort services for delivery. A sizeable majority attended VHSNC meetings and visited households to see newborns. Participation in other activities, such as in managing minor ailments in their villages, being DOTS (Directly Observed Treatment, Short-course) providers for tuberculosis patients and organizing village meetings for health action, however, appeared to be suboptimal.

### Beneficiary reports—healthcare provision by ASHA workers

Table 3 shows that ASHAs were very functional with regard to antenatal and intranatal care service provision, such as counselling support and escort service. At least 60% (689/1,141) of women who had reported an institutional delivery attributed it to being a result of the motivation by the ASHA in their community. There was variability in the type of postnatal care services offered by the ASHA: some activities, such as advice on breastfeeding (83.6%) and home-visits to see the puerperal mother (72.4%), were reasonably high while, on the other hand, service provision on others, such as advice on danger sign management (14.9%), contraceptive-use (21.2%), and maternal nutrition (58.4%), remained low.

Counselling by ASHAs on early initiation of breastfeeding (83.5%) and immunization at birth (84.2%) was reported to be high by the mothers. It was, however, low in other areas, such as exclusive breastfeeding (64.7%), thermoregulation of newborns, including no early bathing (42.4%), keeping baby warm (68.7%), and social determinants of health, such as birth registration (55.8%).

Less than 10% of mothers reported giving pre-lacteal feeds. Only 40% of babies were started on complementary feeding at 6 months of age; about 15% were started prematurely (<5 months), and 45% were delayed (>6 months). In a vast majority (94.5%) of the children, immunization services were facilitated by the ASHA.

A large proportion of mothers reportedly sought ASHA's support for childhood illnesses, like acute diarrhoea (71%) and acute respiratory illness (52.5%). Only about of half the children with diarrhoea, however, received ORS (49.8%) from the ASHA, and half of the children with ARI received any care/advice (52.2%) from ASHA.

### Qualitative study

The reported achievements, gaps, and predominant roles within the three main domains of ASHA's work, as perceived by the officers at various levels, were collated into a matrix as shown in Table 4. Ensuring home-visits and antenatal care, motivation for institutional deliveries (along with providing escort services at the time of delivery), and immunization were listed as key achievements as a link-worker or facilitator. There was scope for improvement in areas, such as postpartum care, newborn care or appropriate referral, infant-feeding (breastfeeding as well as complementary feeding), and assisting with vital event registration. As a community health worker, they saw value in the ASHA being a contact point for general minor ailments but also identified specific gaps in the care of children with diarrhoea or lower respiratory tract infections and also with regard to participation in the tuberculosis control programme. As a social activist, they were organizing or attending VHSNC meetings but were yet to take on key roles in community mobilization for health rights and collective action as also making extra efforts at social inclusion through targeting of marginalized households. Overall, they perceived the role of ASHA to be of mainly a link-worker or facilitator, to a moderate extent as a community health worker and to a small degree as a social activist.

**Table 2.** Self reported effectiveness of ASHAs

Activity	Number (%)	95% CI
Household visits (N=294)	253 (86.0)	82.1-90.0
Maternal and child health		
Counselling women on all aspects of pregnancy	259 (88.1)	84.3-91.8
Accompanying women for institutional delivery	283 (96.3)	94.1-98.4
Visiting newborn for advice/care	215 (73.1)	68.0-78.1
Promotion/coordination for immunization	256 (87.1)	83.2-90.9
Attended/organized VHNDs	170 (57.8)	52.1-63.4
Consultation for childhood illnesses and use of drug kit/referral	120 (40.8)	35.1-46.4
Nutrition counselling	202 (68.7)	63.3-74.0
Infectious diseases		
DOTS provider for tuberculosis patients	176 (59.9)	54.3-65.5
Any malaria control-related work	94 (32.0)	26.6-37.2
Activism/Advocacy		
VHSNC meetings	217 (73.8)	68.8-78.8
Village meetings on health/environment/rights issues	128 (43.5)	37.8-49.1

VHND=Village Health and Nutrition Day

DOTS=Directly Observed Treatment, Short-course

VHSNC=Village Health Sanitation and Nutrition Committee

**Table 3.** Beneficiary-reported effectiveness of ASHAs

Activity	Number* (%)	95% CI
Mothers with children aged <6 months [N=1,141]*		
Women met by ASHA (≥3 times) during antenatal period	967 (84.5)	82.4-86.6
Women escorted by ASHA to a facility for delivery	875 (76.7)	74.2-79.0
Women with institutional delivery and reporting motivation by ASHA	689 (60.0)	57.2-62.8
Women who received advice on breastfeeding from ASHA	954 (83.6)	81.5-85.7
Women visited by ASHA (≥3 times) during postpartum period (6 weeks)	826 (72.4)	69.8-74.9
Mothers with children aged >6 months [N=564]*		
Maternal nutrition	330 (58.4)	54.3-62.5
Care during excessive bleeding	84 (14.9)	11.9-17.8
Contraceptive-use	120 (21.2)	17.8-24.6
Early initiation of breastfeeding	471 (83.5)	80.4-86.6
Exclusive breastfeeding	365 (64.7)	60.8-68.6
No early bathing	239 (42.2)	38.1-46.3
Keeping the baby warm	387 (68.7)	60.8-68.6
Immunization at birth	476 (84.2)	81.2-87.2
Birth registration	315 (55.8)	51.7-59.9
Proportion of mothers meeting ASHA for childhood illness	555 (98.4)	97.4-99.4
Proportion of mothers with a child having diarrhoea during the preceding month and having received services of ASHA [N=228]	162 (71.1)	67.4-74.8
Proportion of mothers with a child having diarrhoea during the preceding month and having received ORS from ASHA [N=228]	113 (49.8)	45.7-53.9
Proportion of mothers with a child with ARI during the preceding month and having received services of ASHA [N=277]	146 (52.7)	48.6-56.8

*Total does not add up to 1,156 and 579 because of missing values

ORS=Oral rehydration salts solution

ARI=Acute respiratory infection

**Table 4.** Achievements, opportunities, and overall role of ASHAs as perceived by the programme officers

Domain	Achievements	Gaps/Opportunities	Overall role
Link-worker/Facilitator	Home-visits Antenatal check-up Institutional births	Postpartum care Newborn care and referral Child-feeding (breastfeeding + complementary feeding)	+ + +
Community health worker	Immunization Contact point for general ailments	Vital event registration Increase ORS-use in diarrhoea Increase care/referral in LRI DOTS-TB provider	+ +
Social activist	VHSNC attendance	Community mobilization for rights and collective action Extra focus on marginalized households	+

+Marginal role

++Moderate role

+++Major role

## DISCUSSION

Overall, the ASHA programme was found to be successfully operational in the villages of the three study districts in Karnataka by adopting a combination of three strategies—demand creation, increased access to services, and local capacity building—that have been documented elsewhere too (18). The sociodemographic profiles of ASHAs were in consonance with what was originally envisaged at the initiation of the programme within the NRHM with a view to getting educated women from the local community with other sources of income for support (19) as community health volunteers. They were broadly representative of the rural populations from which they were drawn in terms of religion, caste, and occupation (11). They were, however, different from health volunteers seen in other settings. They were about eight years younger and were more educated (with over 90% having completed eight years of schooling in our region compared to about 40% of them who had completed five years of schooling in Nepal) (19).

In terms of task performance, it was seen that there were similarities in some domains–over 95% reported advising on antenatal care, and about 50% offered advice on oral rehydration to children with diarrhoea. There were differences, however, in other tasks. While about 72% of volunteers were present with the mothers at the time of delivery in Nepal, over 95% accompanied pregnant women for institutional delivery in our study. On the contrary, while 52% offered advice/care for children with ARIs in our region, over 80% provided such care in Nepal (19).

So, while at the national level in India, there may be differences in the way the ASHA programme was conceptualized and the way the functions within the three domains have been interpreted by the stakeholders, our study offers some insights into the ‘achievements’ and ‘opportunities’ for the ASHA programme that national and state-level programme officers could consider in improving the programme in future. The ASHA workers performed tasks mostly as link-workers and community health workers, and only to a small extent, as social activists. Within the domain of their link-worker role, through their home-visits to the households of community members, they had contributed substantially to improvements in the basic antenatal care check-up programme and also in increasing the proportion of institutional deliveries and immunization coverage (16). There are, however, opportunities for increasing their role in postpartum care (home-visits during puerperal period, counselling on danger signs and prompt referral) for reducing maternal morbidity and mortality (given that about two-thirds of maternal deaths occur during this period) (20). Similarly, targeted home-visits and better counselling on essential newborn care (feeding, temperature regulation), identification of danger signs and early referral for common killers, such as diarrhoea and pneumonia, along with nutritional counselling (appropriate complementary feeding) could help improve neonatal and child health (21). It also offers opportunities for changes within the programme that could be considered by states regionally. Where certain parameters (such as immunization) are successful, there could be diversion of incentives to relatively weaker areas of the programme identified locally so that the programme is flexible at the state level and can be contextualized according to the local needs. Assistance with birth registration could also serve as an important intervention on the social determinants of health (10,22).

As a community health worker, they appear to be the first point-of-contact for several childhood illnesses. However, their role vis-a-vis specific lethal conditions, such as diarrhoea and pneumonia, needs to be specifically elaborated and their training needs to be enhanced to contribute to saving lives. This is especially important given that 80% (111/138) of developing countries are not on track to achieving MDG 4 aimed at reduction in childhood mortality within 2015 (1). Similarly, a more robust mechanism needs to be evolved for replenishment of their drug stocks at the peripheral level. Care for infectious diseases also needs to be improved specifically in the field of tuberculosis where they could contribute to both ‘case-finding’ and ‘case-holding’ (23).

The role as a social activist is the one that is least covered currently. Given their socioeconomic and demographic background and the context in which they work within the hierarchy of the Indian rural community and within the healthcare system, this is understandable. However, special inputs in improving social justice through reducing health inequities at the community level needs to be emphasized as part of their training and routine monitoring and supervision. In addition, skills in assisting with collectivization of the community for public health efforts where individual action may not bear fruit effectively also need strengthening. These are important for several developing-country settings with either existing community health worker schemes (8) or considering new recruitments.

In addition, there is the issue of inadequate coverage of marginalized households within villages and hamlets in rural and peri-urban Karnataka. Special training of ASHAs needs to be undertaken since one of the primary objectives of the ASHA programme was to improve social justice. The importance of key equity stratifiers, such as age, sex, geography, and socioeconomic status for several health outcomes, needs to be emphasized in both training modules as well as in routine supervision (24).

### Conclusions

By and large, functionality of ASHAs in terms of carrying out tasks was reasonably high. Further improvements will require continuous capacity-building to improve knowledge and skills of ASHAs through basic and refresher training as well as mentoring by the *taluk* and district teams, given that they are ‘lay workers’ and not ‘qualified professsionals’. In addition, health system strengthening through provision of drugs and moral support, special skill building in individual, and group motivation for behaviour change targeted to both ‘unfinished agenda’ of communicable diseases, maternal and perinatal conditions, nutritional disorders as well as the newer disease burden due to non-communicable diseases and injuries are critical needs (9,10). The curriculum and inputs need to reflect the ongoing epidemiologic transition in the rural areas of the south India as well (25). Several of these health-promotive and lifesaving interventions are well within the power of ASHAs, if ably supported by the local, state and central governments (26), which may take the support of non-governmental organizations and civil society as needed. Political will and health systems support for the ASHA programme, a key component of the National Rural Health Mission, will go a long way in making a significant change to the lives of millions of residents in this state and elsewhere.

## ACKNOWLEDGEMENTS

The authors acknowledge the support of Dr. Maya Mascarenhas, MYRADA Foundation, Bangalore; Dr. Reynold Washington, KHPT, Bangalore; and Ms Vidya, SHSRC-Karnataka for assistance with field support in data collection.

**Conflict of interest:** Authors declare no competing interests.
